# CCL20/CCR6-mediated migration of regulatory T cells to the *Helicobacter pylori*-infected human gastric mucosa

**DOI:** 10.1136/gutjnl-2013-306253

**Published:** 2014-01-16

**Authors:** Katherine W Cook, Darren P Letley, Richard J M Ingram, Emily Staples, Helle Skjoldmose, John C Atherton, Karen Robinson

**Affiliations:** Nottingham Digestive Diseases Biomedical Research Unit, School of Medicine, The University of Nottingham, Nottingham, UK

**Keywords:** HELICOBACTER PYLORI, T LYMPHOCYTES, CHEMOTAXIS, MUCOSAL IMMUNOLOGY

## Abstract

**Background:**

*Helicobacter pylori*-induced peptic ulceration is less likely to occur in patients with a strong gastric anti-inflammatory regulatory T cell (Treg) response. Migration of Tregs into the gastric mucosa is therefore important.

**Objective:**

To identify the homing receptors involved in directing Tregs to the gastric mucosa, and investigate how *H pylori* stimulates the relevant chemokine responses.

**Design:**

Gastric biopsy samples and peripheral blood were donated by 84 *H pylori*-infected and 46 uninfected patients. Luminex assays quantified gastric biopsy chemokine concentrations. Flow cytometry was used to characterise homing receptors on CD4^+^CD25^hi^ Tregs. *H pylori* wild-type and isogenic mutants were used to investigate the signalling mechanisms behind CCL20 and IL-8 induction in gastric epithelial cell lines. Transwell assays were used to quantify Treg migration towards chemokines in vitro.

**Results:**

CCL20, CXCL1-3 and IL-8 concentrations were significantly increased in gastric biopsy samples from *H pylori*-infected patients. CCR6 (CCL20 receptor), CXCR1 and CXCR2 (IL-8 and CXCL1-3 receptors) were expressed by a higher proportion of peripheral blood Tregs in infected patients. Most gastric Tregs expressed these receptors. *H pylori* induced CCL20 production by gastric epithelial cells via *cag* pathogenicity island (*cag*PAI)-dependent NF-κB signalling. Foxp3^+^, but not Foxp3^−^, CD4 cells from infected mice migrated towards recombinant CCL20 in vitro.

**Conclusions:**

As well as increasing Treg numbers, *H pylori* infection induces a change in their characteristics. Expression of CCR6, CXCR1 and CXCR2 probably enables their migration towards CCL20 and IL-8 in the infected gastric mucosa. Such qualitative changes may also explain how *H pylori* protects against some extragastric inflammatory disorders.

**Significance of this study**

**What is already known about this subject?**

- *Helicobacter pylori* infection is the major cause of peptic ulcer disease and gastric adenocarcinoma.
- Individuals infected with *H pylori* have increased populations of immunosuppressive regulatory T cells (Tregs) in their gastric mucosa.
- Those with an insufficient Treg response tend to have greater levels of inflammation in the gastric mucosa, and are more likely to develop peptic ulcer disease.

**What are the new findings?**

- The concentration of the chemokine CCL20 is dramatically increased in the gastric mucosa of patients infected by *H pylori* and the vast majority of mucosal Tregs express its receptor CCR6.
- Gastric biopsy samples from patients infected with *cag*+ strains contain higher concentrations of CCL20. CCL20 expression is induced in gastric epithelial cells in a *cag* type IV secretion system dependent manner.
- Recombinant CCL20 induces the migration of Tregs in vitro, demonstrating its importance as a chemoattractant for these cells.
- Homing receptor profiles of circulating Tregs differ between infected and uninfected patients. *H pylori* therefore imparts qualitative differences to these populations.

**How might it impact on clinical practice in the foreseeable future?**

- This work indicates the importance of the CCL20–CCR6 axis in the recruitment of Tregs to the gastric mucosa. As this axis is also important in IBD, the study might contribute to the development of new treatments to suppress gastrointestinal inflammation.

## Introduction

*Helicobacter pylori* colonises the stomach in around half of the world's population.^1^ In most cases the infection is established during childhood and persists lifelong.^2^ This chronic infection leads to continuous low-level gastric inflammation, which is asymptomatic in most cases. However, some people develop more pronounced inflammation, which can lead to peptic ulcer disease (PUD) or gastric cancer.^3^ By understanding the factors which influence the inflammatory response to *H pylori* and gastric infiltration of immune cells, we may be able to identify those who are at increased risk of developing disease.

Proinflammatory chemokines and cytokines, such as interleukin 8 (IL-8), are secreted by gastric epithelial cells in response to *H pylori* infection, leading to immune cell recruitment.^2^ *H pylori* expresses virulence determinants, including the polymorphic vacuolating cytotoxin gene A (*vacA*) and the *cag* pathogenicity island (*cag*PAI), which influence disease risk.^1^ The *cag*PAI encodes a type IV secretion system (T4SS), which delivers the effector protein CagA and soluble bacterial cell wall components into host cells.^4^ Inflammatory signalling pathways are activated directly by CagA or through NOD1-recognition of cell wall components.^5^ ^6^ The resulting cytokine production leads to increased mucosal infiltration of neutrophils, macrophages and proinflammatory T-helper subsets including Th1 and Th17 cells and an increased risk of PUD.^5^ ^7^

One of the major modulators of inflammation is the subset of CD4 T cells known as regulatory T cells (Tregs). These cells suppress both innate and adaptive immune responses, and inhibit the activity of T-helper cells.^8^ Previously we showed that CD4^+^CD25^hi^ cells are more abundant in the gastric mucosa of *H pylori*-infected patients, and that poor Treg responses are associated with PUD.^9^ Recruitment of Tregs to the gastric mucosa is important for controlling inflammation and pathology.^9–12^

We previously found that Tregs are also more abundant in the peripheral blood of *H pylori*-infected patients.^13^ This population of Tregs appears to have slightly different characteristics from those found in the gastric mucosa, in that not all peripheral blood CD4^+^CD25^hi^ cells express FOXP3. We hypothesised that some peripheral blood Treg populations in infected patients are programmed for homing to the inflamed gastric mucosa.

The chemokines CCL2, CCL4, CCL5, CCL22 and CXCL8 are known to play a role in the migration of Tregs into inflamed or tumour tissues.^14–16^ Others, including CCL2-5, CCL20 CCL22, CXCL1-3, CXCL8 and CXCL10 are upregulated in the *H pylori*-infected gastric mucosa,^5^ ^17–20^ and expression of chemokine receptors and other adhesion molecules by immune cells is also increased.^10^ ^21^ ^22^ Our first step was to narrow down this array of homing molecules and receptors to those that might be important in Treg migration to the *H pylori*-infected gastric mucosa.

We began this study by quantifying chemokines in gastric biopsy lysates and found that IL-8, CCL20 and CXCL1-3 concentrations were the most upregulated in biopsy samples from infected compared with uninfected patients. We then went on to screen peripheral blood Tregs for a panel of homing receptors. We identified altered profiles of the chemokine receptors CCR6, CXCR1 and CXCR2 on Tregs from infected patients. Next, we defined the signalling pathways involved in *H pylori*-mediated induction of CCL20 expression by gastric epithelial cell lines. Finally, we showed that Tregs from infected mice can migrate towards CCL20 in vitro. These findings show that Tregs can be recruited by the gastric mucosal chemokine response to *H pylori*. This mechanism of Treg recruitment may be important for controlling inflammation and reducing cancer risk among people infected with *H pylori*.

## Materials and methods

Reagents and chemicals were obtained from Sigma-Aldrich (Poole, UK) and fluorochrome-conjugated antibodies were obtained from BioLegend (San Diego, USA), unless otherwise stated.

### Participants and clinical materials

Samples were donated by 180 patients (aged 18–86 years) undergoing routine upper gastrointestinal endoscopy at Queen's Medical Centre, Nottingham, UK (collected 2002–2012) after approval by the Nottingham Research Ethics Committee 2 from an unselected patient group, for whom *H pylori* status could be confirmed and the appropriate clinical materials were available. Patients presented with a variety of indications, most commonly dyspepsia, but were otherwise healthy. None of the patients were regularly taking non-steroidal anti-inflammatory drugs or other immunomodulatory drugs in the preceding 4 weeks, or antibiotics or proton pump inhibitor drugs during the preceding 2 weeks.

Blood samples and a series of eight gastric antral biopsy samples were collected with informed written consent.^9^ Complete sets of blood and biopsy samples were not available for all subjects. *H pylori* status was determined by rapid biopsy urease test, bacterial culture and histology. *H pylori* isolates were *cagA* genotyped as previously described.^23^ Clinical endoscopy observations were recorded. Uninfected donors had no gastric inflammation or disease.

### Luminex immunoassays

Chemokine concentrations were measured by Luminex (see online supplementary methods) in antral gastric biopsy samples from 84 *H pylori*-infected donors (31 duodenal ulcer, 5 gastric ulcer, 48 without ulceration) and 39 uninfected donors and normalised for total protein.^24^ These groups were 52% and 46% male, respectively; mean ages 53.5 and 54.0 years.

### Cell isolation and analysis by flow cytometry

Samples from 35 *H pylori*-infected donors (10 duodenal ulcer, 1 gastric ulcer, 24 without ulceration) and 46 uninfected donors were analysed by flow cytometry. These groups were 64% and 52% male, respectively; mean ages 51.0 and 53.6 years.

Six gastric biopsy samples were collected into culture medium (RPMI 1640/10% fetal calf serum/100 U/mL penicillin G/100 μg/mL streptomycin sulfate), rubbed through a sterile disposable 100 μm cell strainer (BD Biosciences, Oxford, UK), washed and resuspended at 1×10^6^/mL.^9^ Peripheral blood mononuclear cells (PBMCs) were purified by density gradient centrifugation using Histopaque-1077 and resuspended at 1×10^6^/mL.

Extracellular staining using anti-CD4-phycoerythrin (PE)-Texas Red (ECD; Beckman Coulter, High Wycombe, UK) and anti-CD25-PE-cyanin 7 (Pc7) with anti-CD127-PE, anti-CD62L-PE (eBioscience, San Diego, USA), anti-integrin αE-PE (eBioscience), anti-CCR10-PE, anti-CXCR1-PE or anti-intergrin β7-PE (eBioscience) and anti-CCR6-Alexa Fluor 647 (A647), anti-CCR7-A647, anti-CCR9-A647 or anti-CXCR2-A647 was carried out before cells were fixed in 0.5% formaldehyde. FOXP3 Perm buffer set (BioLegend) permeabilised cells were stained with anti-FOXP3-Alexa Fluor 488 (A488). Data on 200 000 events were acquired using a Beckman Coulter Cytomics FC500 and analysed with Weasel V.3.0, using appropriate isotype controls.

### Cell lines and bacterial strains

The human gastric epithelial MKN28 and AGS (ATCC CRL-1739) cell lines were maintained in RPMI 1640 and nutrient mixture F12 Ham medium, respectively, supplemented with 10% heat inactivated fetal calf serum and 2 mM L-glutamine (Invitrogen) at 37°C in a 5% CO_2_ humidified atmosphere.

*H pylori* strains were cultured on Blood Agar Base No 2 containing 5% (vol/vol) horse blood (Oxoid, Cambridge, UK) at 37°C under microaerobic conditions. c*ag*PAI+ *H pylori* strains 60190, 84183,^25^ 26695, B128 7.13 and the *cag*PAI− strains SS1^26^ and Tx30a^25^ were used, along with isogenic mutants deficient in *vacA* (60190ΔvacA, 84183ΔvacA), *cagA* (60190ΔcagA, 84183ΔcagA) and *cagE* (60190ΔcagE, 84183ΔcagE). A *slt* (HP0645) deletion mutant (26695Δslt) and 26695 parental strain were kindly donated by Dr Richard Ferrero, Monash University, Victoria, Australia.^6^

### In vitro culture experiments

24-Well culture plates were seeded with 5×10^4^ cells/well 24 h before experiments. An *H pylori* suspension was added to a multiplicity of infection (MOI) of five bacteria for each epithelial cell, and cultures were incubated for a further 24 h. MOIs were confirmed by viable cell counts. Cell culture supernatants were aliquoted and frozen at −80°C.

Human CCL20/MIP-3α ELISA (R&D Systems, Abingdon, UK) and Human IL-8 CytoSet ELISA (Invitrogen, Paisley, UK) kits were used, with a standard curve on each plate.

### Inhibitor studies and siRNA transfections

Epithelial cells were treated with chemical inhibitors or siRNA duplexes as previously described.^27^ Specific chemical inhibitors—U0126 (10 μM; MEK1 inhibitor), SP600125 (10 μM; c-Jun-N-terminal kinase (JNK) inhibitor), SB203586 (10 μM; p38 inhibitor) and 6-amino-4-(4-phenoxyphenylethylamino) quinazoline (1 μM; NF-κB activation inhibitor)—were added to the cells for 1 h before and throughout 24 h infection. Control cells were treated with 50 ng/mL of recombinant tumour necrosis factor α (TNFα) (PeproTech, Rocky Hill, USA) to activate NF-κB.^9^

Validated siRNA duplexes targeting *NFKB1*, *RELA* and mitogen-activated protein kinase 1 (*MAPK1*) mRNA using HiPerfect transfection regent (QIAGEN, Hilden, Germany) were added to the cells 48 h before 24 h infection. Control cells received HiPerfect only, non-silencing AllStars Hs Negative Control siRNA or AllStars Hs Cell Death Control siRNA. Gene knockdowns were confirmed by western blotting using NF-κB p50 (Cell Signalling Technology Inc, Massachusetts, USA), NF-κB p65 (Merck Millipore, Darmstadt, Germany), extracellular signal-regulated kinase (ERK) (Santa Cruz Biotechnology, Santa Cruz, USA) and anti-actin antibodies.

### Mouse procedures

Animal experiments were approved by the University of Nottingham animal welfare and ethical review body, under UK Home Office Licence 40/3399. Foxp3-green fluorescent protein (GFP) C57/BL6 mice (JAX strain B6.Cg-Foxp3^tm2(EGFP)Tch^/J)^28^ were infected by oral gavage on three alternate days with doses of 1 × 10^9^ *H pylori* strain B128 7.13 in 100 µL Isosensitest broth (Oxoid).^11^ After 3 weeks mice were humanely killed, their spleens were removed and rubbed through a sterile disposable 100 μm cell strainer. Cells were enriched using an EasySep Mouse CD4 T cell enrichment kit (StemCell Technologies, Vancouver, Canada), and sorted into GFP^+^ and GFP^−^ populations using a MoFlo XDP flow cytometer (Beckman Coulter). Infection was confirmed using quantitative culture of stomach tissue homogenate, as described previously.^29^ The median colonisation density for the infected mice was 1.31×10^5^ colony-forming units/g (range 0.625–3.88×10^5^) and colonies were confirmed as *H pylori* by urease tests and Gram staining.

### Migration assay

Migration assays were performed using 96-well Transwell plates with a 5.0 µm polycarbonate membrane (Corning Life Sciences, Corning, USA).^30^ Culture medium (150 μL) with or without 500 ng/mL of recombinant mouse CCL20 was placed in the lower chamber.^31^ Culture medium (100 μL) containing 1×10^5^ cells was placed in the upper chamber. Each experiment was performed in triplicate. After 5 h, migrated cells in the lower chamber were counted. Migration index was calculated by dividing the number of migrated cells by the mean migration seen in the medium-only control wells.

### Statistical analysis

Statistical analyses were carried out using Prism 6.00 (GraphPad Software, California, USA). A two-tailed p value ≤0.05 was taken as significant. In vivo data were displayed in box-and-whisker plots showing median, IQR and total range, and compared using Mann–Whitney U tests. In vitro data were described using means and SDs, and analysed using one-way analysis of variance with Dunnett's post hoc test for multiple comparisons.

## Results

### CCL20, IL-8 and CXCL1-3 concentrations were greatly elevated in *H pylori*-infected gastric biopsy samples

**Figure 1:**
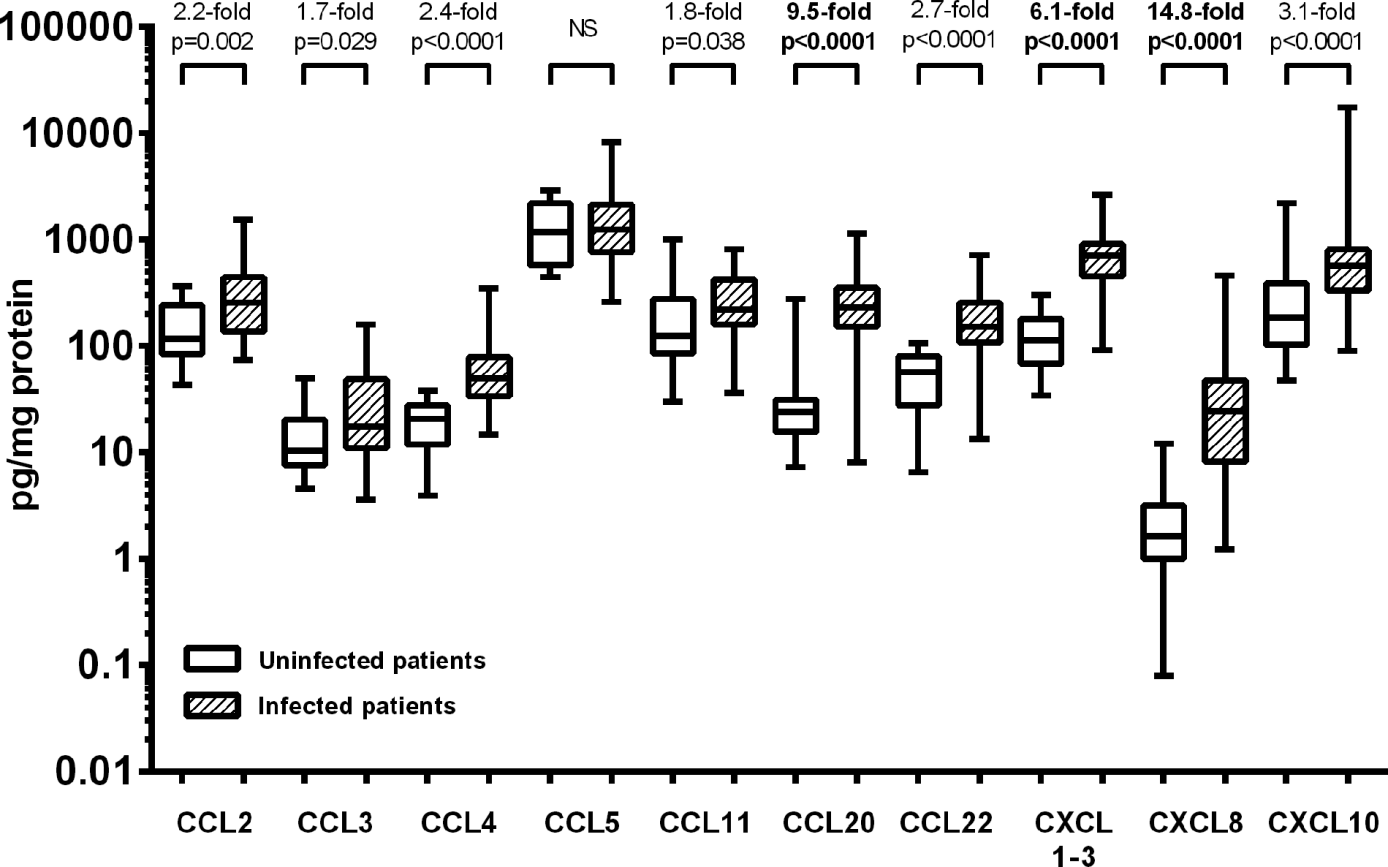
Analysis of gastric chemokine levels from *Helicobacter pylori*-infected and uninfected patients. Gastric biopsy samples from 84 *H pylori*-infected and 38 uninfected patients were disrupted and cytokine levels were determined using Luminex. CCL2 (MCP-1), CCL3 (MIP-1α), CCL4 (MIP-1β), CCL5 (RANTES), CCL11 (Eotaxin), CCL20 (MIP-3α), CCL22 (MDC), homologous cytokines CXCL1-3 (GROα, β and γ), CXCL8 (IL-8) and CXCL10 (IP-10) concentrations were corrected for the total protein content and plotted on a log_10_ scale. p Values denote significantly higher concentrations in biopsies from *H pylori*-infected patients than in samples from uninfected patients, NS, non-significant.

We began by identifying chemokines which were the most upregulated during *H pylori* infection. The chemokine concentrations in gastric biopsy samples from infected and uninfected patients were measured using Luminex assays. Most were upregulated during infection; however, the greatest increases were in CCL20 (MIP-3α), CXCL1-3 (GROα, β and γ) and CXCL8 (IL-8). The median concentrations in biopsy samples from infected patients were 9.5-fold higher for CCL20 (p<0.0001) and 14.8-fold higher for IL-8 (p<0.0001) compared with those from uninfected patients (figure 1). The chemokines CXCL1-3 are >90% homologous and this kit could not distinguish between them; however, we observed that their combined median concentrations were 6.1-fold higher (p<0.0001) in infected patients. Other chemokines were increased by only 1.7–3.1-fold.

### Peripheral blood Treg homing receptor profiles are altered in *H pylori*-infected patients

Having identified the chemokines most upregulated during infection, we examined homing receptor expression profiles on circulating Tregs and whether these were altered in infected patients. We hypothesised that differences in peripheral blood Treg homing receptor profiles in the infected patients might indicate receptors which contribute to Treg migration to the infected gastric mucosa.

**Figure 2:**
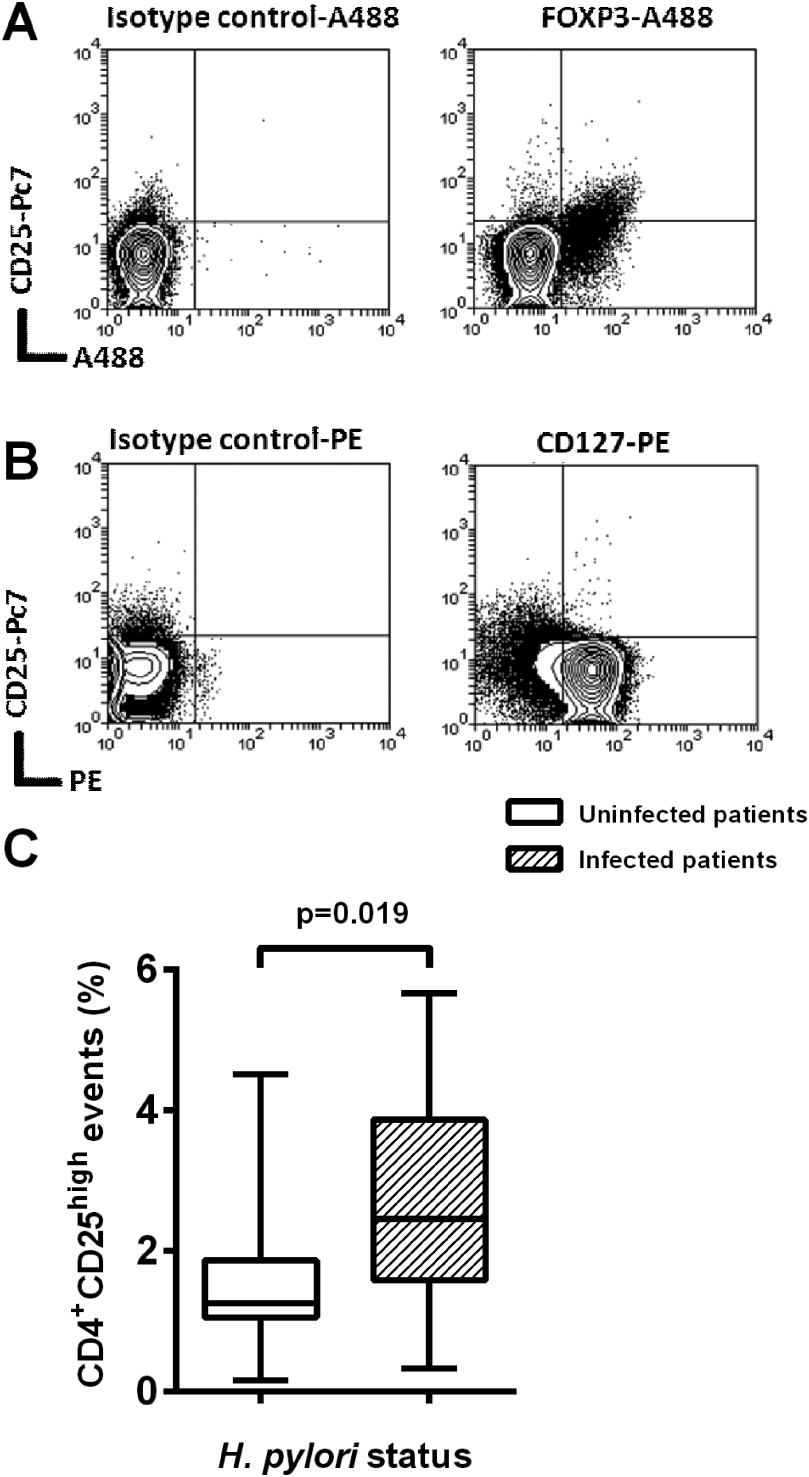
Proportion of CD4^+^CD25^hi^ cells in the peripheral blood of *Helicobacter pylori*-infected and uninfected control patients. Peripheral blood mononuclear cells (PBMCs) from 18 *H pylori*-infected and 20 uninfected patients were isolated, stained and analysed using flow cytometry. Cells were stained with CD4-ECD, CD25-Pc7, CD127-PE and FOXP3-A488 fluorochrome-conjugated monoclonal antibodies. Example plots show CD4^+^ gated PBMCs from one *H pylori*-infected donor demonstrating reproducible CD25^hi^ gating criteria based on (A) FOXP3^+^ and (B) CD127^lo^ events. Isotype controls for A488 and PE are shown. (C) The proportion of CD4^+^CD25^hi^ lymphocytes was compared for infected and uninfected patients.

PBMCs from 18 *H pylori*-infected and 20 uninfected patients were analysed using flow cytometry. Gating on lymphocytes from forward and side scatter plots, Tregs were defined as CD4^+^CD25^hi^ events. The CD25^hi^ gate was set corresponding to the position of CD127^lo^ and FOXP3^+^ events (figure 2A,B). Using these gating criteria, the proportion of CD4^+^CD25^hi^ events among the lymphocyte population was significantly higher in the *H pylori*-infected patients (p=0.02) (figure 2C).

**Figure 3:**
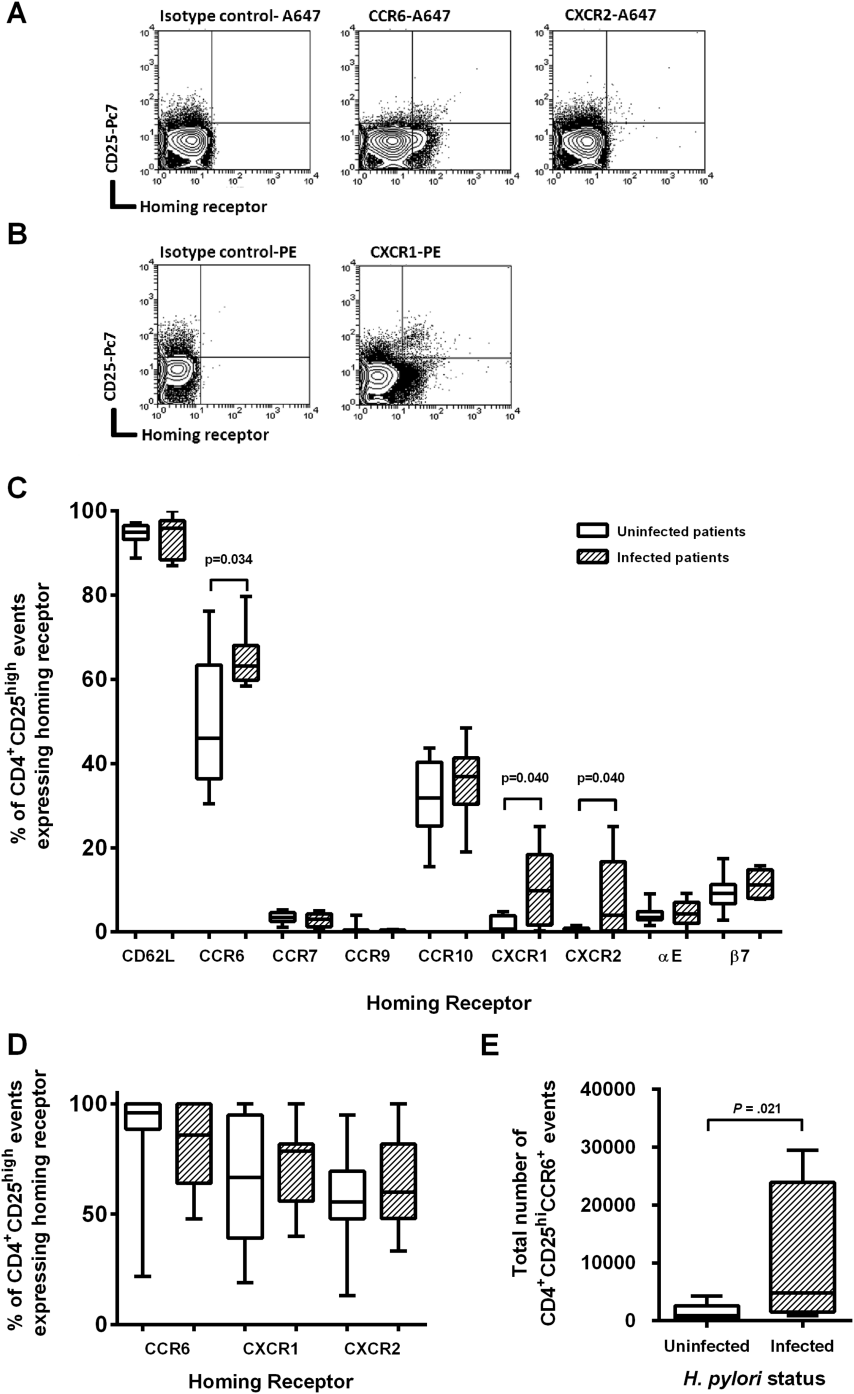
Expression of homing receptors on CD4^+^CD25^hi^ events. Cells were isolated and stained for Treg markers and homing receptors. Example plots show CD4^+^ events with the gating used for (A) CCR6-A647 and CXCR2-A647 and (B) CXCR1-PE staining. The proportion of CD4^+^CD25^hi^ events expressing each receptor was determined using flow cytometry. (C) Peripheral blood mononuclear cells were analysed from 18 *Helicobacter pylori*-infected and 20 uninfected patients and (D) gastric biopsy samples from 18 *H pylori*-infected and 26 uninfected patients were analysed. (E) In addition, the total numbers of CD4^+^CD25^hi^CCR6^+^ events isolated from gastric biopsy samples were calculated. p Values denote statistically significant differences.

PBMCs were then analysed for a panel of homing receptors using flow cytometry (figure 3). The proportion of CD4^+^CD25^hi^ events expressing each homing receptor varied widely (figure 3C). When examining cells from all patients together, a median of 94% of the CD4+CD25^hi^ events expressed CD62L, 57% were CCR6^+^ and 34% CCR10^+^. Less than 10% expressed CCR7, CCR9, CXCR1, CXCR2 or integrins αE and β7. Some receptors varied according to *H pylori* status. Compared with uninfected patients, a significantly higher proportion of CD4^+^CD25^hi^ events from infected patients were CCR6^+^ (medians: 46.7% and 63.2% respectively, p=0.034), CXCR1^+^ (medians: 0.7% and 9.8%, p=0.040) and CXCR2^+^ medians: 0.2% and 4.0%, p=0.040). Given these results we concentrated further investigations on the role of CCR6 (CCL20 receptor), CXCR1 and CXCR2 (IL-8 and CXCL1-3 receptors).

### Most gastric mucosal Tregs express CCR6 and many express IL-8 receptors

We next examined if CCR6, CXCR1 and CXCR2 were also present on gastric Tregs. Gastric biopsy lymphocytes from 18 infected and 26 uninfected patients were stained for CCR6, CXCR1 and CXCR2 and analysed using flow cytometry (figure 3D). Over 90% of the CD4^+^CD25^hi^ events from gastric biopsy samples expressed CCR6, regardless of *H pylori* status. The proportions of CD4^+^CD25^hi^ cells expressing CXCR1 and CXCR2 were also higher in the gastric biopsy samples than the PBMCs (72.6% and 57.8%, respectively), but there was no significant difference between the infected and uninfected patients. The total number of CD4^+^CD25^hi^ and CD4^+^CD25^hi^CCR6^+^ events in biopsies from each patient was calculated (figure 3E). As previously described the number of CD4^+^CD25^hi^ events was increased in infected patients (median 4921) compared with uninfected patients (median 1025; p=0.014). Biopsy samples from *H pylori*-infected patients also contained significantly increased numbers of CD4^+^CD25^hi^CCR6^+^ events (medians 837.5 and 4754, respectively; p=0.021). These results show that Tregs in the gastric compartment are enriched for those expressing CCR6, CXCR1 and CXCR2, and suggest that they may direct Treg homing to the mucosa.

### *H pylori-*induced CCL20 and IL-8 expression by gastric epithelial cell lines is *cag*T4SS dependent

**Figure 4:**
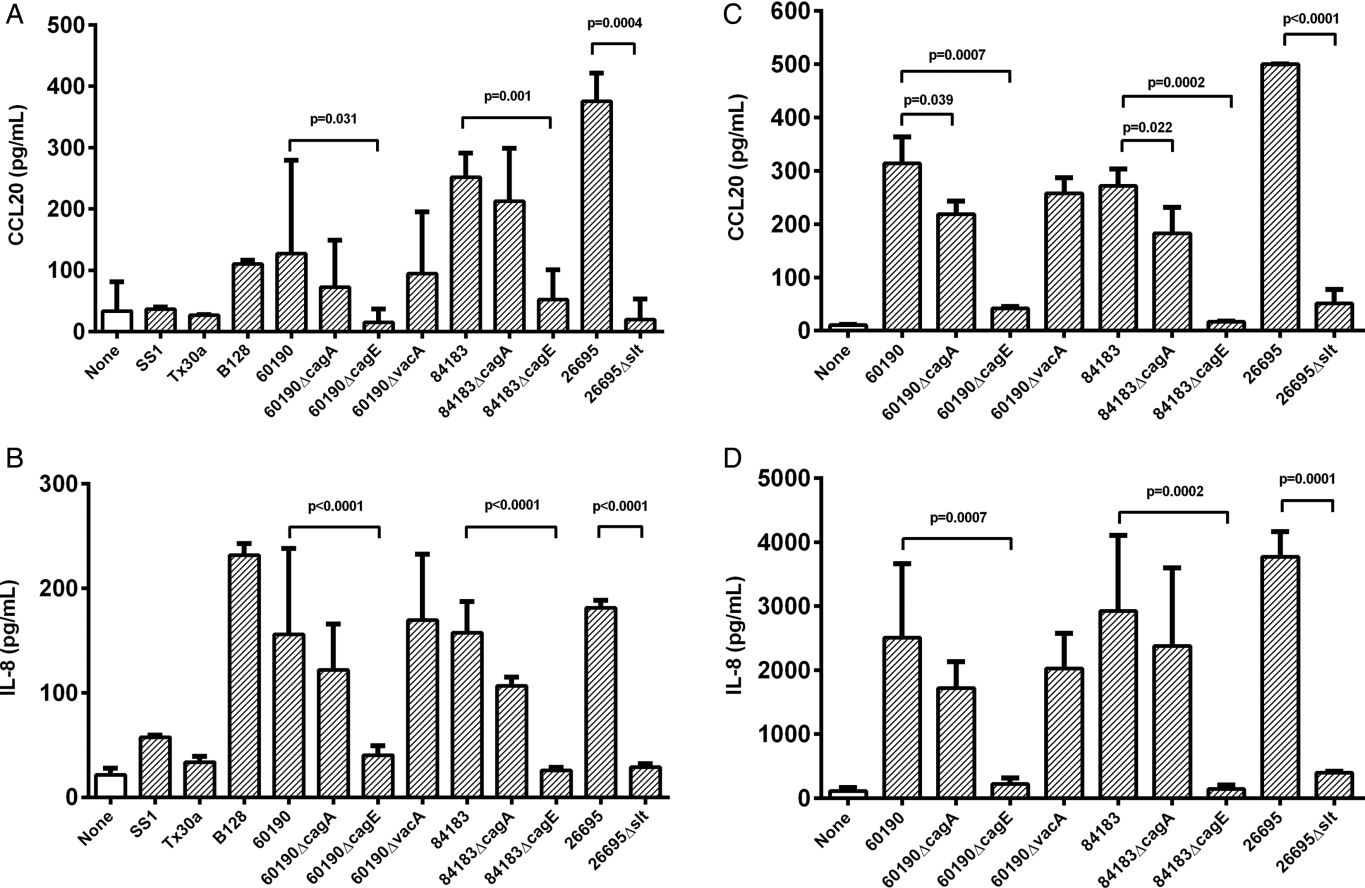
The effect of bacterial virulence factors on chemokine expression in vitro. AGS (A,B) and MKN28 cells (C,D) were co-cultured with *cag*-deficient *Helicobacter pylori* strains Tx30a, SS1 or mutant series constructed in *cag*-positive strains B128, 60190, 84183 and 26695 (MOI=5). After 24 h, CCL20 (A,C) and IL-8 (B,D) concentrations in culture supernatants were measured by ELISA. Graphs show means and SDs from three independent experiments, each performed in triplicate. p Values display significant differences compared with parental strains. MOI, multiplicity of infection.

Next, we used two human gastric epithelial cell lines to examine the role of *H pylori* virulence factors in inducing the CCL20 response in vitro. We also examined IL-8 which, unlike CXCL1-3, is a T cell chemoattractant, and has been shown to be predominantly induced by the *cag*T4SS.^6^ Our biopsy data suggest that CCL20 induction may also be dependent on the *cag*T4SS; therefore IL-8 responses were measured as a control. MKN28 and AGS cells were infected with *H pylori* at a MOI of five bacteria per cell for 24 h before assaying CCL20 and IL-8 production. Compared with uninfected AGS cells, CCL20 concentrations were significantly increased by co-culture with strains 60190 (3.8-fold, p=0.005), 84183 (7.5-fold, p<0.001), 26695 (11.2-fold p<0.0001) and B128 (3.3-fold, p=0.011) (figure 4A). These strains all have an intact *cag*PAI and express the active s1/m1 form of VacA. In contrast, Tx30a (*cag*PAI-negative, s2/m2 *vacA*) and SS1 (defective *cag*PAI, s2/m2 *vacA*) did not significantly stimulate CCL20 production above the level of uninfected controls, but induced low-level IL-8 expression (figure 4B).

Cell lines were then co-cultured with isogenic mutants derived from multiple parental stains of *H pylori*, null for candidate *cag*PAI genes and *vacA*. Their effects on CCL20 and IL-8 were similar, but more pronounced in MKN28 than AGS cells (figure 4). The Δ*cagA* mutants induced less CCL20 than their wild-type parental strains but this was only significant for MKN28 cells (60190ΔcagA:1.4-fold lower, p=0.039; 84183ΔcagA: 1.5-fold, p=0.022). However, deletion of the *cagE* gene, resulting in a disabled T4SS, led to CCL20 concentrations similar to those of uninfected controls for both cell lines (compared with wild-type for AGS cells, 60190ΔcagE: 9.9-fold lower, p=0.031; 84183ΔcagE: 3.3-fold lower, p=0.001). Disruption of *vacA* did not alter the levels of CCL20 production. These data suggest that the *cag*T4SS is necessary for *H pylori*-induction of CCL20, but this is only partially dependent on CagA delivery.

The *cag*PAI also induces IL-8 via peptidoglycan activation of NOD1. To determine if this mechanism is also important in CCL20 induction, cells were cultured with an *slt* null mutant (26695Δslt). This mutant has comparable growth rates to its parental strain, but produces up to 40% less cell wall disaccharide tripeptide owing to a defect in the *slt*-encoded lytic transglycosylase.^6^ ^32^ The 26695Δslt mutant stimulated 9.9-fold (p<0.0001) and 19.1-fold (p=0.0004) lower CCL20 concentrations than the wild-type by MKN28 and AGS cells respectively (figure 4A,C). A similar decrease in IL-8 was also seen, in line with previous reports.^6^ ^25^ These results show that CCL20 expression is induced by *H pylori* in a *cag*T4SS-dependent manner, largely via CagA-independent signalling. In agreement with this, gastric biopsy samples from patients infected with *cagA*+ strains contained higher CCL20 concentrations (p=0.021) than those infected with a *cagA*− strain (see online supplementary figure S1).

### The NF-κB and MAPK1-pathways are important in *H pylori*-induced CCL20 expression

**Figure 5:**
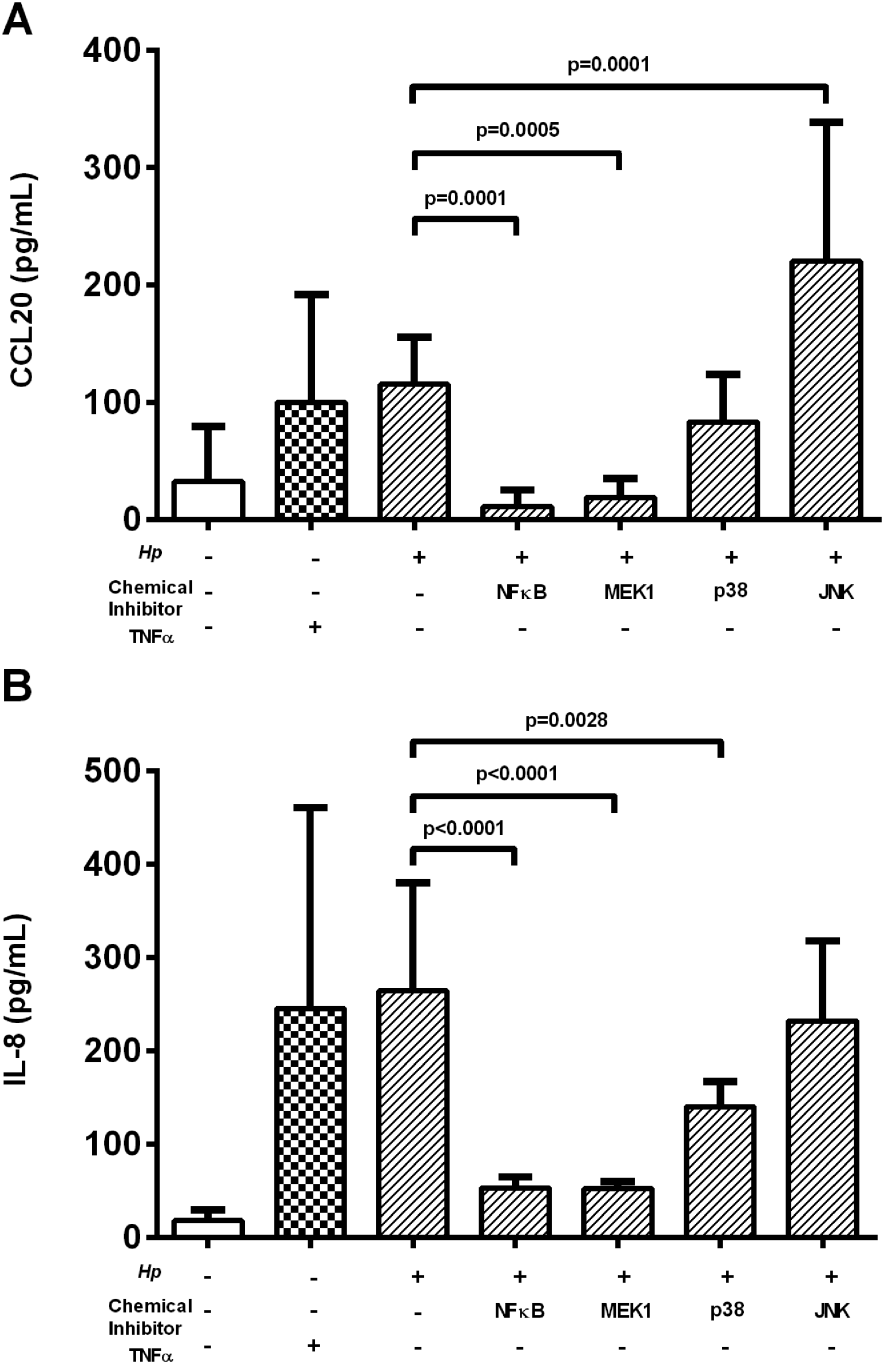
Role of signalling pathways in *Helicobacter pylori*-stimulated CCL20 secretion by epithelial cells. MKN28 cells were treated before and during infection with chemical inhibitors for NF-κB, JNK, MEK1 or p38, or drug diluent alone (see ‘Materials and methods’). Tumour nercrosis factor α (TNFα) treatment was included as a positive control NF-κB inducer. (A) CCL20 and (B) IL-8 supernatant levels were assessed using ELISA. Graph shows means and SDs from four independent experiments performed in triplicate. p Values denote significantly different concentrations compared with *H pylori*-infected controls. JNK, c-Jun-N-terminal kinase.

We investigated the role of NF-κB and the ERK, p38 and JNK MAP kinase signalling pathways, known to be activated by the *cag*T4SS.^4^ MKN28 cells were cultured with *H pylori* strain 60190 in the presence of specific drug inhibitors for each pathway and the effects on CCL20 and IL-8 production were assessed (figure 5). NF-κB and MEK1 inhibitors significantly reduced induction of CCL20 compared with untreated control wells (10-fold, p=0.0001 and sixfold, p=0.0005, respectively), whereas the effect of the p38 inhibitor did not reach statistical significance. JNK inhibition appeared to increase CCL20 expression (1.9-fold, p=0.0001). Uninfected cells were treated with recombinant TNFα as a positive control activator of NF-κB, which increased CCL20 expression (threefold, p=0.018). The treatments had very similar effects on IL-8 expression. These results confirm the importance of NF-κB and suggest the MAPK1 pathways may also have a significant role in *H pylori*-induction of CCL20.

**Figure 6:**
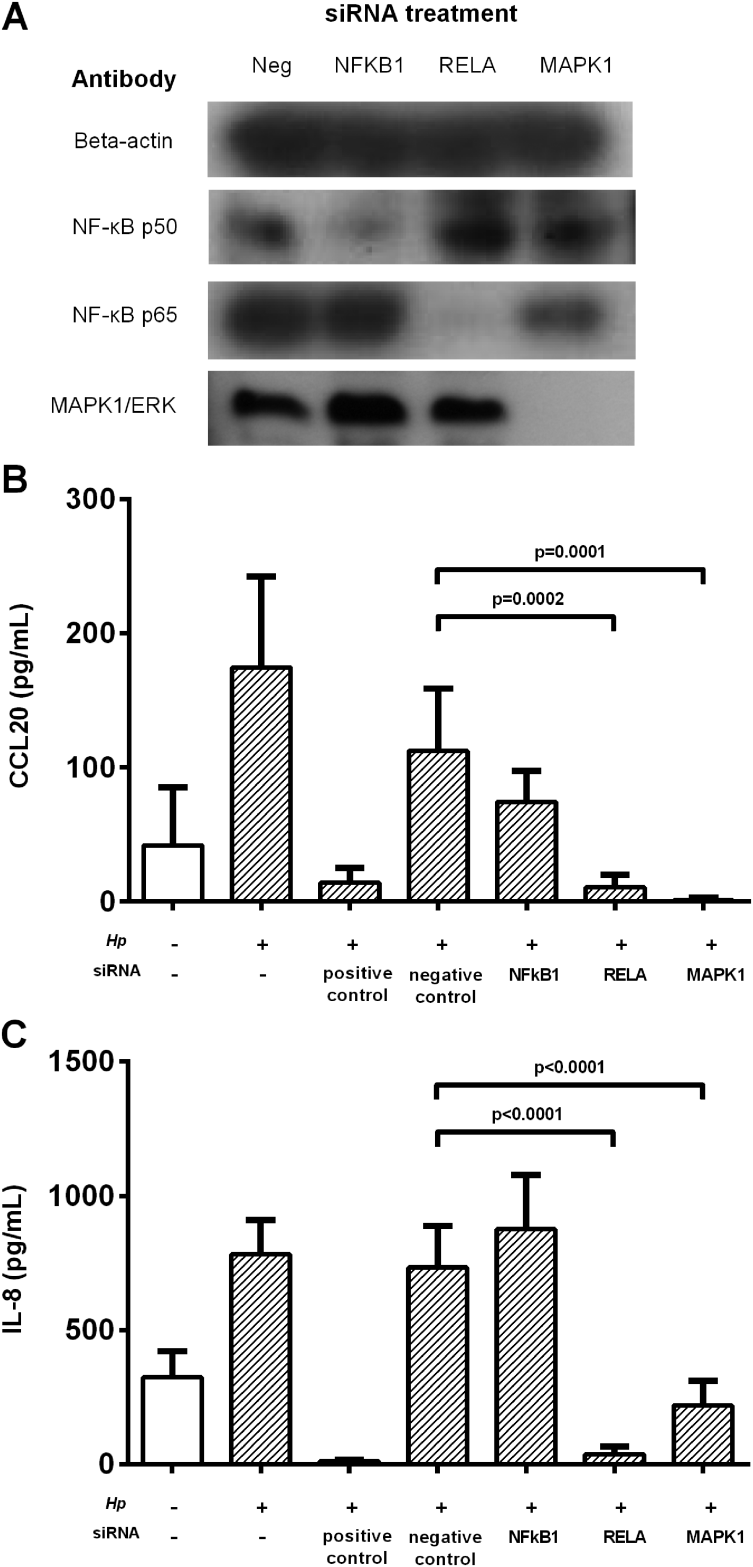
*Helicobacter pylori*-dependent expression of CCL20 by epithelial cells in vitro after siRNA knockdown of signalling pathways. MKN28 cells were pretreated with siRNA duplexes in HiPerfect transfection reagent for 48 h. siRNA treatments targeted NF-κBp50 (NFKB1), NF-κBp65 (RELA) and MAPK1 genes. Negative controls were non-silencing duplexes; positive control duplexes targeted genes necessary for survival. Cells were then treated with 60190 *H pylori* for 24 h. (A) Western blot analysis showing knockdown by siRNA constructs. Supernatant CCL20 (B) and IL-8 (C) levels were then determined using ELISA. Graphs show means and SD from three independent experiments performed in triplicate. p Values show significant differences compared with *H pylori*-infected siRNA negative controls. MAPK, mitogen-activated protein kinase.

To investigate these mechanisms more precisely, MKN28 cells were treated with siRNA duplexes to silence expression of *NFKB1* (which encodes the NF-κBp50 subunit), *RELA* (NF-κBp65) and *MAPK1* for 48 h before infection with 60190 *H pylori* (figure 6A,B). In agreement with the previous data, greatly reduced CCL20 concentrations were seen in infected cells where *RELA* (11-fold, p<0.001) and *MAPK1* (108-fold, p<0.001) expression was knocked down (figure 6B). These duplexes had similar effects on IL-8 expression (figure 6C), suggesting that IL-8 and CCL20 are induced by the same pathways.

### Mouse Tregs migrate towards CCL20

**Figure 7:**
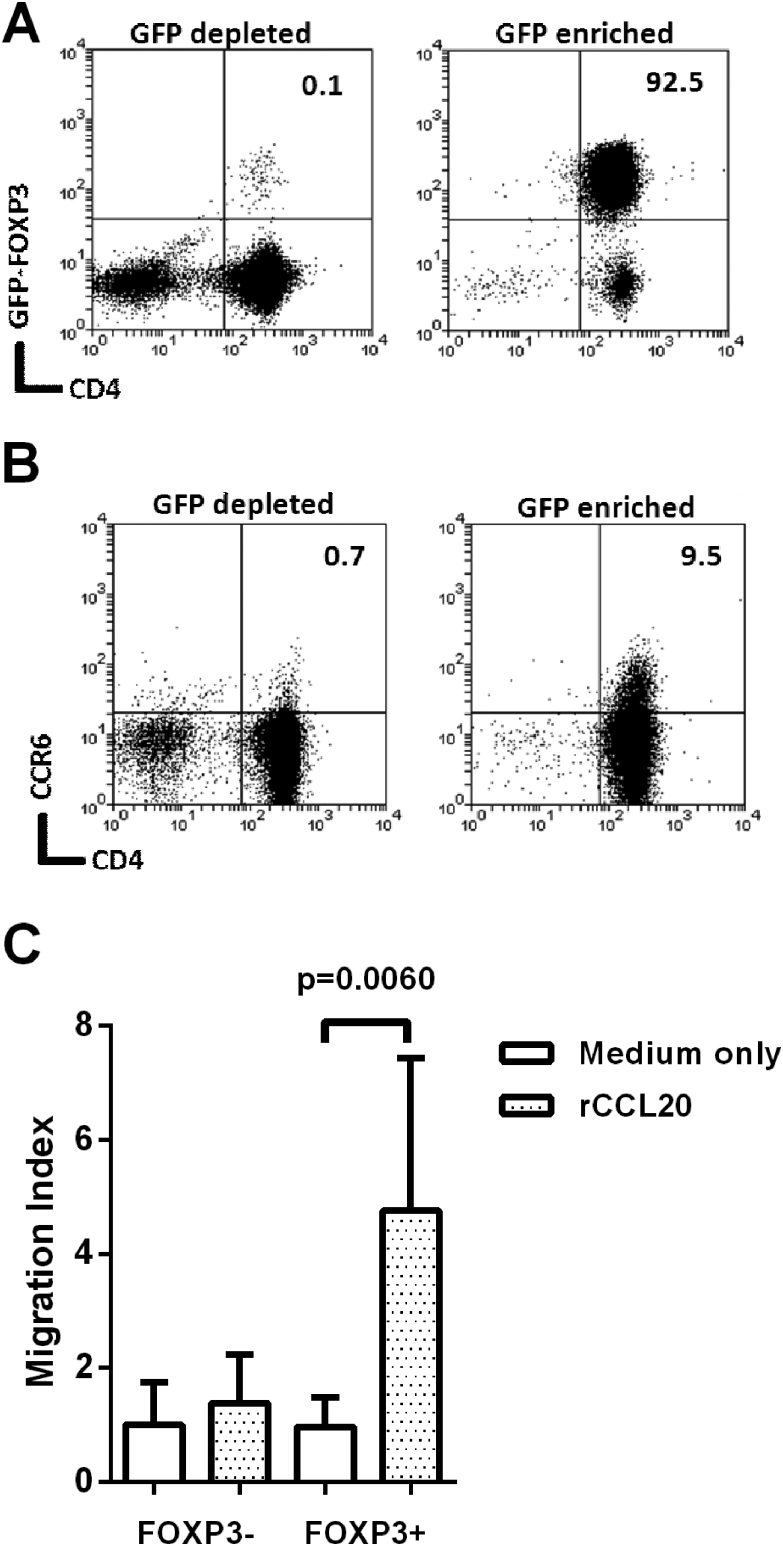
Migration of mouse Foxp3^+^ cells towards recombinant CCL20. Foxp3-GFP C57/BL6 mice were infected with *Helicobacter pylori* strain B128 7.13. After 3 weeks their splenocytes were harvested and pooled. CD4 enrichment was performed using EasySep separation and then GFP^+^ and GFP^−^ populations were sorted using flow cytometry. The CD4^+^FoxP3-depleted fraction and CD4^+^FoxP3-enriched fractions (A) were stained for CCR6 (B). Numbers at the top right denote the percentage of double positive events. Migration of these cells towards recombinant 500 ng/mL of recombinant CCL20 (rCCL20) was assessed using a 96-well Transwell plate (C). The migration index was calculated by dividing the number of migrated cells in the lower chamber of each well, by the average migration seen in the medium-only control wells. The graph shows means and SDs from three independent experiments each performed in triplicate. GFP, green fluorescent protein.

To investigate whether CCL20 is a chemoattractant for Tregs, we used Foxp3-GFP transgenic mice as this provides the most reliable means of Treg cell purification. Mice were infected with the *cag*PAI+ *H pylori* strain B128, and killed 3 weeks later. As expected there were higher frequencies of CD4^+^Foxp3^+^ events in gastric mucosa of infected mice (medians of 0.44% of CD3^+^ events for uninfected and 1.75% for infected, p=0.002). GFP^+^ and GFP^−^ CD4^+^ splenocytes were isolated from infected mice (figure 7A). Flow cytometry analysis showed that 9.5% of the GFP^+^ Foxp3^+^ population expressed CCR6, compared with only 0.71% of the GFP^−^ Foxp3^−^ cells (figure 7B). Migration of these populations towards 500 ng/mL recombinant murine CCL20 (rCCL20) was assessed using an in vitro transwell assay (figure 7C). Foxp3^−^ cells did not migrate; however, the Foxp3^+^ population showed significant migration (p=0.006). This is probably due to the increased proportion of CCR6^+^ events amongst Foxp3^+^ cells.

## Discussion

We found that Tregs in human gastric biopsy samples are predominantly CCR6^+^, which allows them to respond to *cag*T4SS-induced CCL20 expression during *H pylori* infection.

This paper provides a number of new findings. For the first time we have described changes in the profiles of chemokine receptors expressed by circulating Tregs from *H pylori*-infected patients compared with uninfected patients. The association between *H pylori* and increased proportions of CCR6^+^, CXCR1^+^ and CXCR2^+^ Tregs in the bloodstream allowed us to target these receptors and their chemokine ligands in subsequent experiments. Previous work has concentrated on characterising the expression of homing receptors on gastric T cells during *H pylori*-induced disease.^19–21^ Indeed, we found that Tregs isolated from the gastric mucosa are predominantly CCR6^+^. However, characterising the peripheral Treg response to infection provides two advantages: first, obtaining blood samples from patients is less invasive than endoscopy, giving greater potential for larger study groups; and second, more Tregs can be recovered, allowing more detailed characterisation using multiple markers.

The intriguing finding that more CD4^+^CD25^hi^cells express CCR6 in the peripheral blood of *H pylori*-infected patients led us to investigate its ligand, CCL20. We demonstrated that CCL20 levels were increased in the gastric mucosa of *H pylori*-infected patients, in keeping with previous findings.^20^ ^33^ However, we also showed for the first time that CCL20 concentrations were increased in the infected gastric mucosa by a similar degree to the widely studied chemokine IL-8, and CCL20 levels were much higher. The other chemokines were almost all present at higher concentrations in infected tissue, but their levels were increased threefold at most. This highlights the importance of CCL20 in the mucosal response to *H pylori.* The high basal levels of CCL20 in the gastric mucosa goes some way to explain why, even in uninfected patients, most gastric Tregs express CCR6. Surprisingly, almost all Tregs isolated from the gastric mucosa expressed CCR6. However, the number of Tregs infiltrating the gastric mucosa is significantly increased in *H pylori* infection^9^ and here we show that the number of CCR6^+^ Tregs in the gastric mucosa of *H pylori* infected patients is also significantly increased.

We built on previous co-culture experiments which showed that CCL20 mRNA levels in gastric epithelial cell lines were reduced in response to a *cag*PAI-deficient strain,^33^ to demonstrate for the first time that peptidoglycan components delivered by the *cag* T4SS are the major inducers of CCL20 production in gastric epithelial cell lines. A number of different transcription factor binding sites have been described in the CCL20 gene promoter region, including sites for activator protein 1 (AP-1) and NF-κB.^34^ MEK and NF-κB signalling have been linked to CCL20 induction in response to proinflammatory stimuli in other cell types, including colonic epithelial cells.^35^ We show that in gastric epithelial cells *H pylori* peptidoglycan components activate ERK and NF-κB signalling, thus inducing CCL20 in a similar way to that already described for IL-8.^6^ Previously, the p38 pathway was shown to be important in activation of IL-8 production in response to *H pylori* peptidoglycan.^36^ Our data confirm that IL-8 is significantly reduced in cells treated with a p38 inhibitor. However, although we observed that inhibiting the p38 pathway led to a consistent reduction in CCL20 production across the three independent experiments performed, this did not reach statistical significance when all data were combined. Therefore, ERK and NF-κB signalling (and possibly also p38) mediate the induction of CCL20 in response to *H pylori* peptidoglycan via the *cag*T4SS, In support of these findings, we observed the highest mucosal CCL20 concentrations in patients infected with a *cagA*+ strain of *H pylori*.

Our results suggest that bacterial strains better equipped to interact with their host, such as those with an intact *cag*T4SS, may more strongly influence the immunoregulatory response. In support of this, we previously showed that patients infected with *cag*PAI+ *H pylori* strains had higher levels of *IL10* mRNA and increased infiltration of CD4^+^CD25^hi^ cells in their gastric mucosa.^9^ These data may appear to be in conflict with the evidence that PUD is more common among individuals infected with *cag*PAI+ strains.^1^ Kido *et al*^37^ have suggested that the *cag*T4SS has a dual role in *H pylori* infection, acting to both promote and suppress gastritis. Using mouse models, they showed that infection, particularly with a CagA+ *H pylori* strain, was important for inducing the migration of T cells (primed by either CagA+ or CagA− infections) into the gastric mucosa, resulting in gastritis. Conversely, adoptive transfer of T cells first primed by a CagA+ infection led to an accumulation of Tregs in the gastric mucosa of recipient mice and gastritis was attenuated; this did not occur with T cells primed by CagA− *H pylori*. Their conclusion that the *cag*T4SS is extremely important for the induction of a protective Treg response is also supported by our findings. In patients infected with a *cag*PAI+ strain, high cytokine expression leads to gastritis, and we propose that the effect of the *cag*T4SS on Treg migration becomes more important for controlling this inflammation. Ulcers probably result when there is a deficiency in this process.

Treg migration patterns contribute to the tight control of their anti-inflammatory effects and can be altered during an ongoing response, to target immune suppression.^22^ ^38^ ^39^ The altered human Treg homing receptor profiles seen here may be important in controlling the inflammatory response to *H pylori*. Tregs reduce inflammation to avoid excess damage to the host and, in *H pylori* infection, inflammation drives disease. Looking at Treg homing might help to identify patients at risk of developing more extensive disease. CCR6 is also expressed by a number of other cell types including dendritic cells and B lymphocytes,^40^ which also play important roles in the host response to *H pylori* infection. CCR6 is highly expressed on the proinflammatory Th17 cell type.^30^ The balance between Th17 and Treg cells is thought to be important in determining the severity of *H pylori-*mediated gastric inflammation.^41^ Both Th17 and Tregs preferentially localise to mucosal surfaces through CCL20 signalling, and the CCL20–CCR6 axis is currently the subject of a great deal of research in IBD.^42^ ^43^ Similar to our findings, CCL20 expression is elevated in the IBD mucosa, and protection from colitis has been shown to be mediated by CCR6^+^ Tregs.^44^ ^45^ There is growing evidence that *H pylori* infection is negatively associated with IBD,^46^ ^47^ but if this is the case the mechanism remains unclear. Induction of CCR6^+^ Tregs by *H pylori* may result in their migration towards CCL20 expressed in the stomach and the intestine to modulate inflammation. Future work therefore needs to elucidate the role of the CCL20–CCR6 axis in the *H pylori-*infected gastric mucosa, and its possible influence on IBD.

In summary, we showed that increased proportions of Tregs express the chemokine receptors CCR6, CXCR1 and CXCR2 in the peripheral blood of *H pylori*-infected individuals. However, the majority of gastric Tregs express CCR6. In gastric biopsy samples CCL20, the only chemokine recognised by CCR6, is increased in response to *H pylori* and is more abundant than IL-8. CCL20 expression is induced in gastric epithelial cells in a *cag*T4SS-dependent manner by *H pylori* via the NF-κB and MAPK pathways. Recombinant CCL20 induces migration of purified mouse Tregs. We are now developing mouse model assays to further characterise the role of the CCL20–CCR6 axis in Treg cell homing in vivo.

## Supplementary Material

Web supplement
