# Supplementary material for: CCL20/CCR6-mediated migration of regulatory T cells to the Helicobacter pylori-infected human gastric mucosa
Source: Gut. 2014 Jan 16;63(10):1550–9. doi: 10.1136/gutjnl-2013-306253 (PMC4173663; doi:10.1136/gutjnl-2013-306253)
Supplement: Web supplement [file gutjnl-2013-306253-s1.pdf]

Supplementary Material

Materials and Methods

Luminex immunoassays

Staples and Ingram et al. previously reported the optimization of this method[24]. Snap frozen gastric biopsies were transferred to 300 μl of ice-cold PBS (pH 7.4, Dulbecco A, Oxoid, Basingstoke, UK) supplemented with 2 mM Mg^{2+} (Sigma-Aldrich), benzonase endonuclease to reduce viscosity (Novagen, Darmstadt, Germany) and EDTA-free cOmplete mini protease inhibitors (Roche, Basel, Switzerland). These were disrupted on ice using a pellet pestle with cordless motor (Kimble Kontes, NJ, USA) for 2 min, homogenized by repeated aspiration through a 200 μl filter pipette tip, and incubated on ice for at least 5 min. Clarified supernatant was obtained by centrifugation at 10,000 × *g* for 10 min at 4 °C, then aliquoted into Protein LoBind microcentrifuge tubes (Eppendorf, Hamburg, Germany) and stored at -80 °C until analysis.

Samples were analyzed using MILLIPLEX MAP Human Th17 Magnetic Bead Panels (HTH17MAG) for MIP-3α (CCL20), High Sensitivity Human Cytokine Magnetic Bead Panels (HSCYTMAG) for IL-8 (CXCL8) and Human Cytokine/Chemokine Magnetic Bead Panels (HCYTOMAG) for MCP-1 (CCL2), MIP-1α (CCL3), MIP-1β (CCL4), RANTES (CCL5), eotaxin (detects eotaxin-1, CCL11), MDC (CCL22), GRO (detects α/β/γ, CXCL1-3) and IP-10 (CXCL10) (Merck Millipore). Assays were run as per manufacturer's instructions with 25 μl (HTH17MAG and HCYTOMAG) or 50 μl (HSCYTMAG) of sample in duplicate, overnight incubation with shaking (18 hours, 4 °C, 750 rpm), and using a hand-held magnetic block (Merck Millipore) for wash steps. Data were acquired on a validated and calibrated Bio-Plex 200 system (Bio-Rad Laboratories, CA, USA) and analyzed with Bio-Plex Manager 6.1 software (Bio-Rad Laboratories) as per manufacturer's instructions with double discriminator (DD) gates of 8000-23,000. Sample wells for individual analyte replicates with bead counts ≤20 were invalidated. Standard curves were fitted using five-parameter logistic regression

with default weighting (power law variance), optimized by the software, and then reviewed. Lower and upper limits of quantification (LLOQ and ULOQ) were determined as the highest and lowest fitted standards with recovery 80-120 percent and CV <20 percent. Where the reported result for an analytes fell outside of this quantifiable range, the appropriate limit was substituted. The mean LLOQs (in pg/ml) were 1.23 for CCL20, 0.10 for CXCL8, 1.58 for CCL2, 10.20 for CCL3, 1.69 for CCL4, 1.39 for CCL5, 9.90 for CCL11, 9.46 for CCL22, 9.95 for CXCL1-3, and 6.12 for CXCL10. Total protein was quantified using a BCA assay kit (Pierce, IL, USA) with a single set of BSA standards, and chemokine concentrations reported in pg per mg protein.

Supplementary Figure Legend

Supplementary Figure 1: Further analysis of gastric chemokine levels from *H. pylori*-infected and uninfected patients.

Gastric biopsies from 107 patients (84 *H. pylori* infected) were disrupted and cytokine levels were determined using Luminex. (*A*) CCL20 and IL-8 concentrations were corrected for the total protein content and plotted on a $\log_{10}$ scale according to the *cagA* status of colonizing strains (40 *cagA*+). (*B*) Biopsy CCL20 and IL-8 concentrations from each patient were plotted. Data were analysed using a Spearman correlation test.

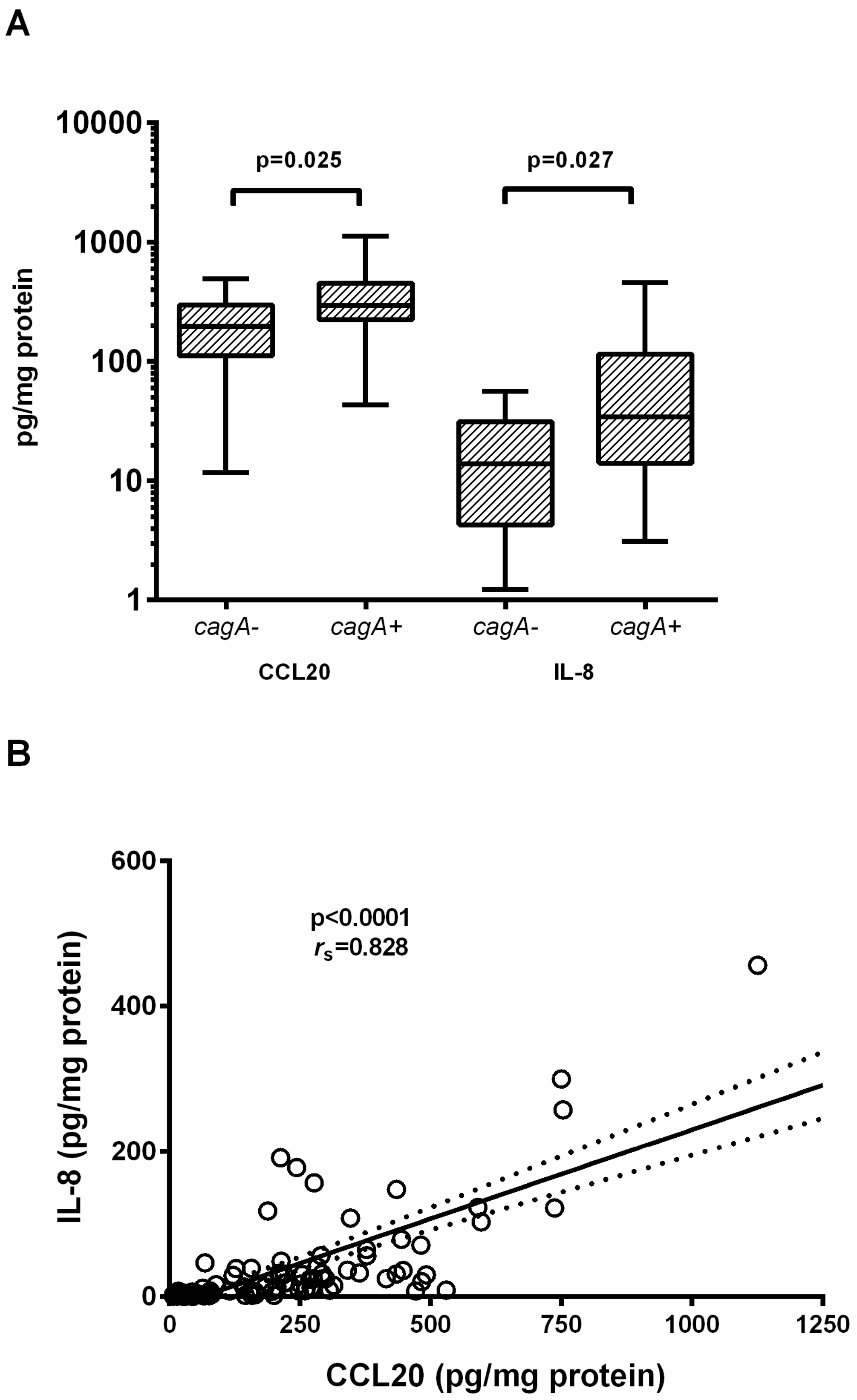
A
p=0.025
p=0.027
10000
1000
100
10
1
pg/mg protein
cagA-
cagA+
cagA-
cagA+
CCL20
IL-8
B
600
400
200
0
p<0.0001
rs=0.828
IL-8 (pg/mg protein)
0
250
500
750
1000
1250
CCL20 (pg/mg protein)
